# Online media reporting of suicides: analysis of adherence to existing guidelines

**DOI:** 10.1192/pb.bp.115.052761

**Published:** 2017-04

**Authors:** Michael Utterson, Jason Daoud, Rina Dutta

**Affiliations:** 1 South London and Maudsley NHS Foundation Trust; 2 King's College London

## Abstract

**Aims and method** To assess the compliance of contemporary online media output with guidelines for the responsible reporting of suicidal acts. A search engine was used to identify online media reports of suicide from UK sources over the course of 1 month. Each article was assessed against guidelines for the responsible reporting of suicide produced by the Samaritans, a UK mental health charity.

**Results** We identified 229 articles, of which 199 failed to comply with at least one of the Samaritans' guidelines. Failure to mention support sources, excessive detail about the method used and undue speculation about the trigger for suicide were the most commonly breached guidelines. Significant differences were found between the quality of local and national media sources, with local media sources being broadly more compliant with guidelines.

**Clinical implications** This study highlights the urgent need for the implementation of responsible reporting guidelines in online media articles as a component of suicide prevention efforts.

Responsible media reporting of suicide is considered to be key to current international suicide prevention efforts.^1^ Previous studies suggest that media reports of completed suicide can influence the rate of future suicidal acts and the method chosen.^2–6^

Guidelines to promote responsible reporting practices exist in a number of countries.^7^ Studies suggest that they can alter the content of media reports,^8^ although the general awareness and use of the guidelines has been found to be poor.^9^ In the UK, the 2012 Editors' Code of Practice from the Press Complaints Commission prohibits the reporting of suicide with excessive detail about the method used,^10^ but established responsible reporting guidelines tend to include additional features in an attempt to prevent copycat behaviour.^11^

After a detailed literature review, no studies could be found comparing a significant number of contemporary UK media reports of suicide against responsible reporting guidelines, despite the fact that the media have been implicated in the phenomenon of ‘suicide clustering’ observed in the UK.^12^ Other studies have focused on newspaper and television reports, and scant attention has been paid to online media sources, despite the fact that 84% of households in the UK have access to the internet and 55% of adults use the internet to access news.^13^ This is the most rapidly growing method of accessing news media, particularly for younger individuals,^13^ who may be more vulnerable to the content of media reporting.^14,15^

This study sought to evaluate the compliance of online media reports of suicidal acts from UK-based sources with responsible reporting guidelines, and to identify the prevalence of the inclusion of other potentially harmful features of online media, namely comments sections and links to other internet features, for example pro-suicide websites.

## Method

A search was performed on the Google News UK search engine using the keyword ‘suicide’ with the location filter set to include articles of UK provenance only. The analysis included reports which made reference to an attempted or completed suicide published by local and national media sources during 28 consecutive days in November 2014. Articles about suicide bombing and euthanasia were excluded, as were those behind a media paywall.

Each report meeting the inclusion criteria was assessed against an adapted version of the ‘Ten things to remember when reporting suicide’ contained in the document issued by the Samaritans.^16^ This is one of the more prominent and widely used sets of guidelines for journalists in the UK.^17^ As a direct search for articles was undertaken, it was not possible to assess whether each article was given undue prominence, for instance a homepage splash, so this criterion from the Samaritans' guidelines was not used, leaving nine criteria for article comparison (Box 1).

Data were collected on compliance with each section of the guidelines and those meeting all criteria were considered to be compliant with responsible reporting of suicide. Analysis was undertaken to determine the overall compliance with guidelines, the frequency with which each guideline was breached, and whether there were any differences based on whether articles originated from a local or a national media source. Differences between local and national media sources in the proportion of articles breaching any guidelines were calculated using the chi-squared test. Data analysis was performed in STATA 11 for Windows XP.

**Box 1** Guidelines for the reporting of suicide assessed in the study. Adapted from the Samaritans^16^

1. Leave out technical details about the method of suicide, such as describing the type of ligature used or the number and types of pills taken in an overdose. Never suggest that a method is quick, easy, painless or certain to result in death.
2. Language matters. Avoid dramatic headlines and terms such as ‘suicide epidemic’ or ‘hot spot’.
3. Include references to support groups and places where suicidal people can find help.
4. Treat social media with particular caution and refrain from mentioning websites or networks that promote or glamorise suicide.
5. Avoid dramatic or sensationalist pictures or video. Refrain from including content from suicide notes.
6. Do not give undue prominence to photographs of a young person who has died and avoid repeated use of images such as galleries.
7. Do not brush over the complex realities of suicide and its impact on those left behind.
8. Speculation about the ‘trigger’ for a suicide, even if provided by a close family member, should be avoided.
9. Use statistics with caution. Make sure you have the most recent data and are comparing like with like.

In addition to comparison against the Samaritans' guidelines, the inclusion of user-generated comments, threads and internet links to other articles was noted.

## Results

Overall, 229 articles met the inclusion criteria: 68 articles from local media sources and 161 articles from national media sources. The majority (214 articles) came from media organisations which offer both a print and online platform, with national publications such as the *Guardian*, the *Telegraph* and the *Daily Mail* making up the bulk of national media output, and an array of smaller local outlets each contributing a smaller number of articles to the total. Fifteen articles were found in a range of online-only outlets such as the International Business Times, Yahoo UK, The Huffington Post and Wales Online. Ten articles were found on the websites of media network providers such as the BBC, ITV and STV.

### Compliance with the responsible reporting guidelines

Of the 229 online articles included for analysis, 199 (86.9%) breached at least one of the Samaritans' guidelines. The mean number of guideline breaches per article was 2.2, with only a small variation between local and national media sources (2.1 *v.* 2.2 breaches per article, respectively; *P* = 0.08).

The most commonly breached aspects of the guidelines were a failure to include reference to sources of support for those considering suicide (69.4%), the inclusion of excessive technical detail about the method used (31%) and undue speculation about the reasons for suicide (30.1%) (Table 1). The other guidelines were breached in less than 25% of articles, with just 2 articles mentioning organisations that promote suicide and 1 article using statistics irresponsibly, telling readers the proportion of people completing suicide after jumping from a well-known landmark.

**Table 1:** Compliance with reporting guidelines

	All sources	Local	National	Difference between local and national *P*
Articles, *n*	229	68	161	–

⩾1 breach, *n* (%)	199 (86.9)	55 (80.9)	144 (89.4)	0.08

Breaches per article, mean	2.2	2.1	2.2	–

Specific guideline breaches, *n* (%)				

1. Excessive technical detail about the method	71 (31.0)	25 (36.8)	46 (28.6)	0.22

2. Sensationalist or irresponsible language	38 (16.6)	20 (14.7)	28 (17.4)	0.04*

3. No sources of support	159 (69.4)	48 (70.6)	111 (68.9)	0.81

4. Mentioning places that promote or glamorise suicide	2 (0.9)	0 (0)	2 (1.2)	0.36

5. Dramatic pictures, videos, content of suicide notes	41 (17.9)	6 (8.8)	35 (21.7)	0.02*

6. Picture galleries	30 (13.1)	1 (1.5)	30 (18.6)	0.001**

7. Narrative brushes over the complex realities of suicide	20 (8.7)	5 (7.4)	15 (9.3)	0.63

8. Undue speculation about the triggers	69 (30.1)	18 (26.4)	51 (31.7)	0.43

9. Irresponsible use of statistics	1 (0.4)	0 (0)	1 (0.6)	0.52

* *P* < 0.05,

** *P* < 0.01.

### Additional features of online media

Sixty-four articles included additional features which could contribute to readers encountering unsuitable material, such as the inclusion of user-generated comments sections and links to other articles which may similarly be poorly adherent to reporting guidelines (Table 2).

**Table 2:** Articles with additional adverse features

	All sources *n* (%)	Local *n* (%)	National *n* (%)	*P*
Articles with additional adverse features	64 (27.9)	11 (16.2)	53 (32.9)	*0.01

Links to other articles about suicide	37 (16.2)	5 (7.4)	32 (19.9)	*0.02

User-generated comment threads	39 (17)	7 (10.3)	32 (19.9)	0.08

* *P* < 0.05.

## Discussion

This study evaluated the compliance of 229 online reports of suicide with the guidelines issued by the Samaritans. To our knowledge, this is the first study looking at the content of online media reports of suicide and the first in the UK looking at a selection of contemporary media output. Of note, the majority of articles included in the study failed to meet the Samaritans' guidelines for the responsible reporting of suicide. The results support findings from the limited number of studies undertaken elsewhere in the world that suggest media reporting of suicide is poorly compliant with available guidelines.^18–21^

Of particular concern is the finding that 69.4% of reports failed to include a reference to a potential source of support for those readers who may be experiencing suicidal thoughts themselves; this was consistent across local and national sources. Lack of responsible information awareness and signposting support undermines suicide prevention efforts and fails to provide an alternative perspective to the often distressing narrative of articles. The finding that most articles omit references to support groups has been replicated in other studies from around the world: just 1% of Indian newspaper sources,^19^ 3% of US sources^21^ and 8.6% of Chinese sources^8^ included a reference to a support group.

Our finding that 31% of articles contained an excessive level of detail about methods used to complete a suicide is a significant cause for concern. The experience in other countries supports the idea that publication of suicide methods can perpetuate attempts and trends in methods chosen by others in ‘copycat suicides’.^2–5,22^ In the course of assessing media reports for this study, the inclusion of details about the blood concentration of cyanide to achieve death, and the exact location and time of suicides by train, as well as details of places where suicides by jumping were completed, were all noted.

The finding that 30.1% of articles engaged in speculation about the reason for suicide was also a cause for concern. The journalistic tendency to simplify the reasons behind a suicidal act or engage in undue speculation about the surrounding circumstances can have a damaging impact on the bereaved family^17^ as well as readers who may over-identify with the person mentioned in the article, potentially increasing the deleterious impact for vulnerable individuals. This tendency to make articles more ‘readable’ may also be reflected in the fact that 8.7% of articles brushed over the complex realities of suicide, often failing to mention the family left behind or the impact of the suicidal act on others.

That being said, only one report included the irresponsible use of statistics and only two reports directed readers towards pro-suicide websites.

A qualitative observation was that where a particular fact about a suicide attempt is known, it will usually feature in other articles from other outlets about the same act. For example, very specific details about a method used were usually re-reported in all articles discussing the same event without due regard for the reporting guidelines.

### Local *v.* national media sources

When comparing reporting by national and local media sources, local sources were overall more compliant with guidelines, with significant differences in the use of sensationalist language, dramatic pictures, videos or the content of suicide notes and the use of galleries, as well as the use of additional features of online media. The exact reason for the broadly better compliance with guidelines among local sources is not fully understood, but it may be because local media sources are closer to the subject of the article and local reporters may be more sensitive to the feelings of the bereaved family and local community.

### Additional features of online media

The unique additional features of online media (compared, for example, with newspaper articles or television reports) could also compound their negative impact on readers; 16% of articles included links to other reports of suicide. Our finding that a majority of articles about suicide fail to meet responsible reporting guidelines and that the mean number of guideline breaches is 2.2 per article raises the possibility that the negative impact of irresponsible reporting is likely to be amplified by the inclusion of links to other potentially non-compliant reports.

Previous findings that discussion forums can increase suicidality among younger users^23^ suggest that the addition of comments sections which can facilitate discussion should be avoided with online reports of suicide. Despite this, 17% of analysed reports had a comments section for user-generated content, and concerning comments such as the deceased person being ‘brave’ or ‘at peace now’ were frequently a feature of these.

### Policy considerations

There is an evident need to evaluate the reasons for journalistic non-compliance with the existing guidelines of suicide reporting in the UK. Given the increasing use of online media and the apparent poor quality of reporting, there is a need to focus efforts on increasing the compliance of reports with responsible reporting guidelines. Suitable measures should also be established for non-compliant and potentially harmful articles to be flagged for urgent review.

### Limitations

Although a standardised tool was used to identify breaches of media guidelines, judgements about breaches were not cross-checked between researchers. In addition, although the search sought to capture publications over a period of time, this work cannot account for potential seasonal changes in data.

### Next steps

The present study uncovers an urgent need to address the fact that the majority of online articles assessed do not comply with existing guidelines on the responsible reporting of suicide. It highlights a significant public health concern because potentially vulnerable people have access to material which may provoke suicidal behaviours and which does not signpost them to support resources. Given the increasing weight of evidence that media reporting can affect suicide rates, there is an urgent need for the implementation of responsible reporting guidelines in online media articles. We propose that work be done to clarify and publicise the guidelines, and to train and encourage journalists to use them, and that a strong consideration be given to the role of more formal regulation and monitoring.
